# Geometric Optimization of Microfabricated Silicon Electrodes for Corona Discharge-Based Electrohydrodynamic Thrusters

**DOI:** 10.3390/mi8050141

**Published:** 2017-05-03

**Authors:** Daniel S. Drew, Kristofer S. J. Pister

**Affiliations:** Berkeley Sensor and Actuator Center, University of California, Berkeley, CA 94720, USA; ksjp@berkeley.edu

**Keywords:** electrohydrodynamic force, corona discharge, atmospheric ion thrusters, flying microrobots, ionocraft

## Abstract

Electrohydrodynamic thrust is an emerging propulsion mechanism for flying insect-scale robots. There is a need to both minimize the operating voltage and maximize the output force when designing microfabricated electrodes for use in these robots. In this work, an array of hybrid wire-needle and grid electrode geometries were fabricated and characterized to attempt to minimize both corona discharge onset voltage and thrust loss factor. Statistical analysis of this dataset was performed to screen for factors with significant effects. An optimized emitter electrode decreased onset voltage by 22%. Loss factor was found to vary significantly (as much as 30%) based on collector grid geometric parameters without affecting discharge characteristics. The results from this study can be used to drive further optimization of thrusters, with the final goal of providing a path towards autonomous flying microrobots powered by atmospheric ion engines.

## 1. Introduction

Insect-scale robots may one day serve as multifunctional swarm agents, coordinated assembly tools, and disposable mobile sensors [1]. Recent work on flying insect-scale robots (or “pico air vehicles” [2]) has focused primarily on biomimetic propulsion mechanisms, i.e., the motion of flapping wings similar to an insect of the Diptera order. Significant progress has been made, including: controlled flight of a flapping wing robot with a piezoelectric actuator [3]; takeoff of a robot with an electromagnetic actuator [4]; and measured thrust using an electrostatic actuator [5]. Nevertheless, biomimetic fliers are difficult to design, build, and control. To open up the design space beyond that afforded by these biomimetic structures it may be necessary to change not just the actuator but the propulsion mechanism itself.

**Figure 1:**
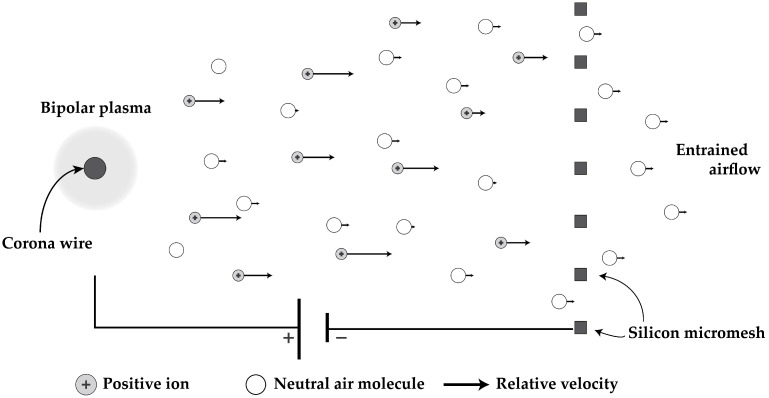
In a positive corona discharge, the “emitter” is biased positively and the “collector” is typically grounded. The resultant electric field in this case will be locally enhanced around the wire as a function of geometry. If the field strength is high enough for electrons to initiate Townsend avalanche breakdown, a local plasma (the corona) will be generated around the emitter. Positive ions, typically $N_{2}^{+}$, are ejected from the plasma and drift under the influence of the electric field towards the collector. Along the way they impact neutral air molecules and produce a net momentum transfer and resultant air flow. Note that the ratio of ions to neutral molecules has been greatly exaggerated in this cartoon.

Electrohydrodynamic (EHD) thrust results from the collisions between a neutral fluid and charged particles. Directional momentum transfer is produced by controlling the motion of the charged particles electrostatically, directly converting electrical energy into kinetic energy. The momentum transferring collisions producing the EHD force are more efficient when using ionized molecules instead of electrons due to their higher mass. The induced air flow from ion-neutral collisions has been called the “ion wind”. The effect was noted as early as 1709 and investigated by minds including Benjamin Franklin, Faraday, and Maxwell [6]. A thorough review on the history as well as other applications of EHD force can be found in [7]. Corona discharge is the preferred method of producing ions for EHD thrust in atmospheric conditions because it has been found to be relatively repeatable, stable, and high current [8]. Design and fabrication are simplified by the fact that corona discharge is predominantly due to geometric enhancement of local electric field. A schematic illustrating the use of a self-sustained corona plasma to produce EHD force is shown in Figure 1.

The EHD force was investigated for use in aircraft in the early 1960s, even being featured on the front cover of *Popular Mechanics* in August 1964. At the time of the *Popular Mechanics* article, the most successful demonstration of EHD thrust was with the “lifter” model now popular with students and hobbyists. The lifter is on the order of 0.1 to 1 square meter, typically constructed from balsa wood and aluminum foil, and can take off when connected to a high voltage (30 kV) power supply. After important fundamental research from investigators including Christenson, Moller, and Robinson, the effect was deemed to have too low of a thrust aerial density and too low of an efficiency for human-scale flight [9,10]. More recent theoretical work on a one dimensional model has seemingly confirmed their conclusions that electrohydrodynamic thrusters are not well suited for these types of applications [11]. We stipulate that EHD is a viable propulsion mechanism not for macro-scale flying aircraft but for flying microrobots, for many of the same reasons that electrostatic motors have proven themselves useful in MEMS-based devices and walking microrobots. As an essentially electrostatic force, the EHD force per unit area remains constant as the device decreases in size.

**Figure 2:**
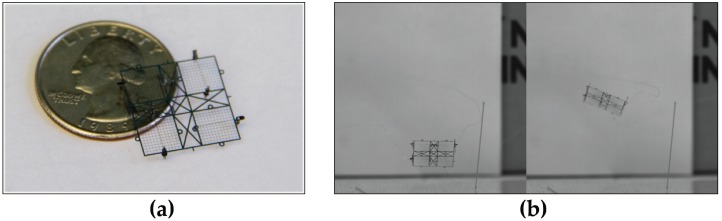
We have recently reported on the use of electrohydrodynamic thrusters for flying microrobots (ionocraft) [13]. The current study is focused on decreasing the ionocraft operating voltage and increasing thrust to weight ratio, and therefore payload capacity, to provide a path towards autonomy. (**a**) A 1.8 cm by 1.8 cm hand-assembled microrobot with four individually addressable thrusters from that work; (**b**) Again as demonstrated in prior work, at about 2500 V it can take flight; a takeoff is shown in two panels taken from a high speed video recording.

EHD thrust as a flight mechanism for pico air vehicles has the unique advantages of silent flight and high durability due to requiring no moving parts, as well as high thrust to weight ratio due to requiring only sparse electrodes. Microfabricated thrusters generating over 10 N/m^2^ with thrust to weight ratios over 20 have been demonstrated [12]. A flying microrobot with four individually addressable EHD thrusters (see Figure 2) has demonstrated vertical takeoff and rudimentary attitude control while tethered to an external power supply [13]. These devices exhibit onset voltages around 2400 V and maximum thrust points closer to 3000 V. In order to present a clear path towards autonomy this operating voltage will have to be reduced to levels feasibly supplied by a combination of low mass boost circuitry, e.g., as shown in [14], and arrays of batteries and/or solar cells. The largest current driver of miniaturization of corona discharge-based EHD devices is for thermal management applications (IC cooling). A review of this research can be found in [15]. It should be noted that millimeter-scale devices are rare. Ong et al. demonstrated microfabricated devices with onset voltages around 1.1 kV, but their low current makes them unsuitable for thruster applications [16]. The important metric of thrust to weight ratio is also unique to thruster applications and is relatively unexplored in literature.

### 1.1. Electrohydrodynamic Force Scaling

A one dimensional model for electrohydrodynamic thrust based on the Coloumb electrostatic force on a volume of ions leads to an expression for force in terms of ion current, *I*, distance the ions travel *d*, and the ion mobility, *μ*. Here *V* is the applied potential and *A* is the ion volume cross-sectional area. This assumes all ion current is due to applied drift field as opposed to bulk fluid motion, an assumption [9] proved accurate for the 3 m/s maximum air velocities measured in this work versus ion velocity that is on the order of 100 m/s.

(1) $$F=\int \rho EdV=\int \rho EAdx=\int \frac{Id}{\mu VA}\frac{V}{d}Adx=\frac{Id}{\mu }$$

This represents the theoretical maximum force from an EHD thruster. Previous work measured loss factors of around 50% from this theoretical value using microfabricated silicon electrodes [12].

Based on the Mott-Gurney law for space charge limited current density, (2), this equation for electrostatic force on the ions can instead be expressed in terms of applied drift field *E* and air permittivity *ϵ*_0_:

(2) $$\begin{array}{cc} I & =\frac{9\epsilon_{0}\mu AV^{2}}{8d^{3}} \end{array}$$

(3) $$\begin{array}{cc} F=\frac{Id}{\mu } & =\frac{9\epsilon_{0}}{8}AE^{2} \end{array}$$

Note that this is force acting on the ions; direct translation of this to thrust requires that all momentum is transferred in the preferred thrust direction by ion-neutral collisions. Assuming applied voltage is the same as the drift field potential and that ion current is the only current flow, this yields a thrust efficiency in terms of *N*/*W* of:

(4) $$\begin{array}{c} P=IV=\frac{9}{8}\epsilon_{0}\frac{A}{d^{3}}\mu V^{3}\\ \frac{F}{P}=\frac{d}{\mu V}=\frac{1}{\mu E} \end{array}$$

These equations indicate that unlike many flapping wing designs, an EHD thruster powered microrobot does not depend on a resonant system and can vary its thrust and thrust efficiency simply by changing the drift field magnitude. In practice, the minimum drift field value for a corona discharge-based system is set by the corona inception voltage. They also show that both the force and efficiency scale inversely with ion mobility. Prior work has shown that the mobility of positive ions is lower than that of negative ions for electrode gaps below 20 mm [17]. Literature values for positive ion mobility in air range from 1.37 to 2.8 cm^2^·V^−1^·s^−1^. This wide range of seemingly acceptable values makes it difficult to generalize results across the field of corona discharge devices, as the true value is also dependent on factors such as ambient humidity, electrode gap, field strength, temperature, and pressure.

### 1.2. Corona Discharge Scaling

The derivations given above for electrohydrodynamic force have assumed some concentration of ions injected into the drift field gap. As noted above, the most common method of atmospheric ion generation for EHD thrust is through the atmospherically-stable corona discharge phenomenon. Corona discharge is characterized by a self-sustained plasma localized around a charged conductor, the “emitter”, and ionic current flowing to a second electrode, the “collector”. The magnitude of the discharge is typically space-charge limited, with the polarity determined by whether high voltage is applied to the so-called emitter (“positive corona discharge”) or the collector (“negative corona discharge”) [18].

In a positive corona discharge, when the potential gradient around the emitter is high enough due to a combination of applied voltage and geometric effects, an ambient electron initiates Towsend avalanche breakdown. At some distance away from the emitter the Towsend ionization criterion is no longer met, marking the corona plasma boundary and the beginning of the ion drift region. Because collisions in the ionization region do not strongly contribute to the net thrust of the system, it is important to minimize the ratio of the ionization region radius to drift distance.

There are no widely reported wholly analytical solutions for corona discharge current. An equation of the form shown below, sometimes referred to as “Townsend’s relation”, has been experimentally confirmed over a wide range of geometries and is assumed true for space-charge limited discharges such as corona [9].

(5) $$I=CV(V-V_{crit})$$

where *V_crit_* is the corona inception voltage and *C* is a constant with units of A/V^2^ that depends on a combination of device geometry and ion mobility. Analytical values for *C* exist for a few simple geometries, e.g., concentric cylinders, but are typically extracted from experimental data.

Peek provided solutions for the critical surface field, derived from the Townsend ionization criterion and empirical data, to initiate corona discharge for various geometries [19]. Using the method of image charges to adapt Peek’s solution of the critical field between parallel wires for a wire and grounded plane, we find the critical potential for corona onset as:

(6) $$V_{crit}=g_{0}r\left(1+0.301/\sqrt{r}\right)ln\left(2d/r\right)$$

where *d* is the distance between the electrodes, *r* is the wire radius, and $g_{0}$ is the bulk breakdown field strength in air (approximately 30 kV/cm). This equation is widely used in literature and consistently produces predicted voltages accurate to within about 10%. The $0.301/\sqrt{r}$ term was empirically derived by Peek. He states further that the quantity $0.301\sqrt{r}$ is approximately equal to the ionization region thickness, being the distance away from the conductor surface at which the electric field is equal to the bulk breakdown field strength. Because the electric field is a function of both radius and distance it is clear that this relationship, which has no distance term, cannot hold true for all values of the ratio d/r. The portion of Equation (6) which does take into account the gap is from a simplified version of the wire to plane electric field equation which assumes *d* is much larger than *r*. Both the full and simplified version of this equation are shown below:

(7) $$\begin{array}{cc} E(x) & =\frac{2V\sqrt{d^{2}-r^{2}}}{\left(r+x\right)\left(d-r\right)-\frac{x^{2}}{2}ln(\frac{d}{r}+\sqrt{{\left(\frac{d}{r}\right)}^{2}-1})} \end{array}$$

(8) $$\begin{array}{cc} E(x) & =\frac{2V}{\left(dx\right)-\frac{x^{2}}{2})ln\left(\frac{2d}{r}\right)} \end{array}$$

Electric fields in the vicinity of the emitter wire as calculated by (8) versus the full version (7) can deviate by as much as 50% for d/r ratios less than 20. These shortcomings may help explain why research exploring sub-millimeter corona discharge with d/r ratios on the order of 15 have seen greater divergence from Peek’s formulas [20]. While Peek stated that corona discharge would occur at d/r ratios down to about 3, they (and others) have seemingly been unable to measure corona discharge before sparkover below a ratio of about 10. This work will investigate methods of decreasing apparent *r* without the use of exotic materials or complicated processing steps in order to continue corona scaling to smaller gap sizes.

We assert that the ratio of ionization region to electrode gap distance is a critical factor to control in order to maximize EHD force. The fundamental electrostatic force given in (1) is proportional to ion drift distance; if the ratio of ionization radius to gap distance is significant, then simply using the electrode gap to determine theoretical force is incorrect. This ratio is typically small for high d/r ratio devices and is safely ignored. However, Vuhuu and Comsa experimentally measured ionization radii on the order of hundreds of micrometers [21], which would be a significant fraction of a sub-millimeter discharge.

### 1.3. Collector Electrode Effects

Previous work has noted that collector electrode geometry significantly affects both corona current and the resultant flow rate, with grid electrodes performing better than rings [22]. Literature on airflow through micromeshes on the order of those studied here is sparse. O’Hern and Torcyznski measured drag coefficients on the order of 1-5 from photoetched meshes with wire widths of 50 μm, thickness of 50 μm, and separations of 318 μm at similar Reynolds numbers to those expected in EHD microrobots [23]. Computational values for drag coefficient from this work were found to be sensitive to mesh wire cross section and experimental results showed a strong dependence on wire separation, with drag coefficient decreasing by about 50% for a 20% reduction in open area fraction.

A further consideration is how the micromesh geometry affects the electric field profile of the device. Maxwell claimed that at gap distances equal to or greater than the grid separation the grid will look virtually identical to a solid plane at the emitter wire [24]. If this holds true for corona discharge devices, decreasing gap distance will necessitate a decreased grid separation and therefore presumably higher drag coefficient.

## 2. Materials and Methods

### 2.1. Electrode Design and Fabrication

Devices are fabricated in a two mask silicon-on-insulator process and individually plasma diced, as described in [13]. The corona emitter wires have nominal cross sections of 40 μm by 40 μm. The silicon resistivity ranges from 1 to 100 Ω·cm.

**Figure 3:**
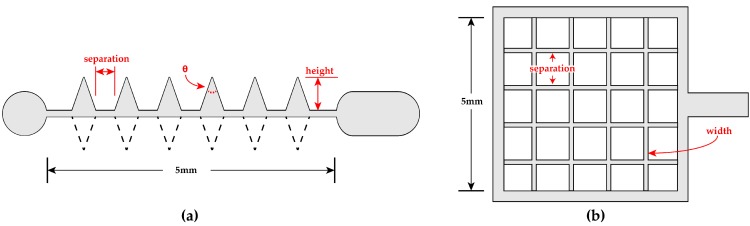
(**a**) The emitter wires have triangular protrusions (“spines”). The distance between spines, height, and tip angle are all varied. Some wires are created with spines only along one half of the wire, while others are fully populated. In all cases the cross section of the rectangular portion of the wire is 40 μm by 40 μm; (**b**) Silicon collector grids have varied separation and wire width. The grids are designed to keep the total interior area constant at 25 mm^2^. Because the wire width is also controlled, the separation is scaled around the set point to accomplish this. In all cases the thickness of the grid is 40 μm.

Emitter wires with periodic protrusions of various shapes and collector grids with varying wire separation and width were fabricated. Figure 3 depicts both of these types of geometries. Table 1 shows the range of swept features. They include tip angle, spine separation and height, and whether the wire is populated with spines on one or both sides.

### 2.2. Characterization Setup

**Figure 4:**
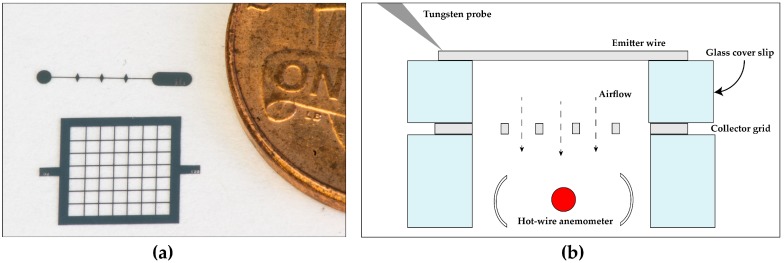
(**a**) A pair of fabricated electrodes next to a U.S. one cent coin for scale; (**b**) A schematic view of the characterization setup. The entire setup is contained within a probe station. Distance between the electrodes is set by glass cover slips. The electrode set is elevated off the probe station chuck by glass spacers so that the conductive chuck does not interfere with measurements and so that the hot wire anemometer can be placed underneath the outlet. Not shown is the second tungsten probe tip which makes electrical contact with the collector grid and the third tungsten probe tip which makes mechanical contact with the other side of the wire to keep it stable during testing.

A Gamma High Voltage supply with 10 kV maximum voltage and 500 μA maximum current is used for electrical testing. A HP benchtop multimeter with a resolution of 100 nA is used for current measurements. A TSI AVM430 hot wire anemometer with a resolution of 0.01 m/s is used for air velocity measurements. The edge of the anemometer inlet is placed within a millimeter of the grid, while the actual hot wire is approximately 2 mm away axially. The anemometer inlet width is about 5 mm, meaning it should collect the full grid outlet airflow without needing to take measurements at multiple points along the channel. A depiction of the test setup is shown in Figure 4. Velocity measurements are internally filtered by the anemometer using a moving average with five second time constant. During testing both the current and velocity were allowed to settle (between 5 and 10 s) before recording the data point.

Output force is calculated using simple momentum theory, where $F=\dot{m}v_{e}=\rho Av_{e}^{2}$. The entire interior area of the collector grid (5 mm × 5 mm) is used as the outlet area. This assumes a negligible inlet velocity and a uniform outlet velocity ($V_{e}$). Previous EHD research showed severe overestimation of thrust by using outlet air velocity as opposed to a scale [17], noting that a single velocity profile is insufficient to accurately calculate the volumetric force. We expect this problem to be less significant both for thrusters of the scale shown here, where the outlet is on the order of the measurement apparatus inlet, and for our test setup, where the outlet flow is ducted by the dielectric standoffs. Previous research has shown fair agreement between mass flow calculations and more direct methods of thrust measurement for similar electrode arrangements [12].

## 3. Results

The presented data is from a range of fabricated devices. Statistical analysis was performed using JMP by SAS Institute.

### 3.1. Corona Inception Voltage and Current

A total of 21 different electrodes were characterized. In all cases a 500 μm grid separation was used. Electrodes were exchanged when catastrophic failure occurred (see Figure 11).

Corona inception voltages and *C* coefficient were calculated by plotting Equation (5) in terms of I/V. This method, as opposed to the method of finding the critical voltage with a μA transition, was deemed more reliable given the current resolution of the test setup. The *C* coefficient can be used as a rough quality factor when designing for high current discharge devices as long as the breakdown voltage does not decrease significantly between electrodes. $R^{2}$ vales above 95% were found in the linear fit for all cases.

**Figure 5:**
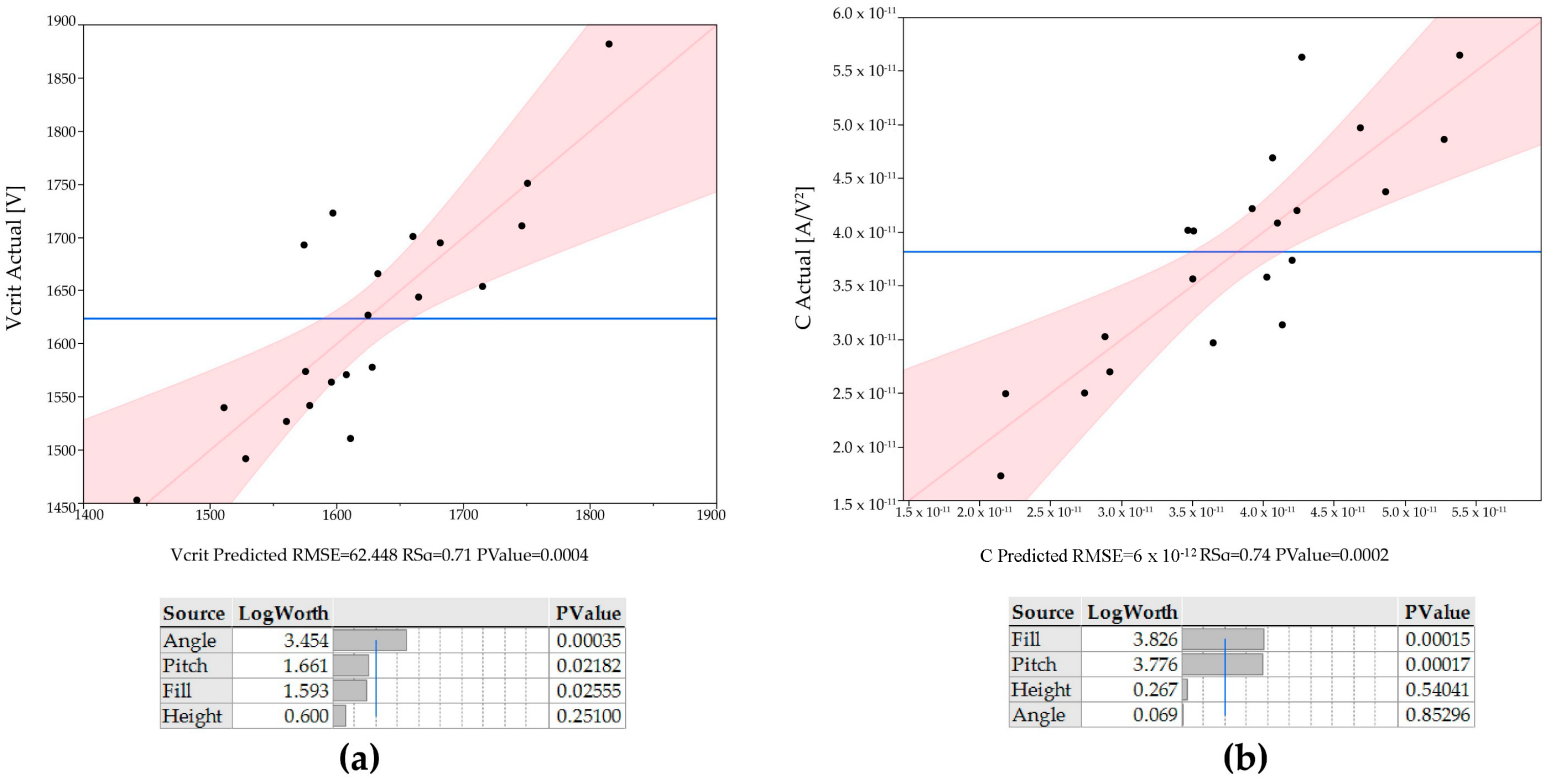
Response models generated by least squares regression analysis of the 21 run dataset. A *p* value below 0.05 generally represents a statistically significant factor (rejection of the null hypothesis). (**a**) The regression model for corona inception voltage; (**b**) The regression model for *C* coefficient.

Commercial software was used for modelling significance of the varied factors on two effects, $V_{crit}$ and *C*. An ideal electrode has a low inception voltage and a high *C*. The dataset was fit to the models using a standard least squares regression analysis. The results of these fit are shown in Figure 5.

**Figure 6:**
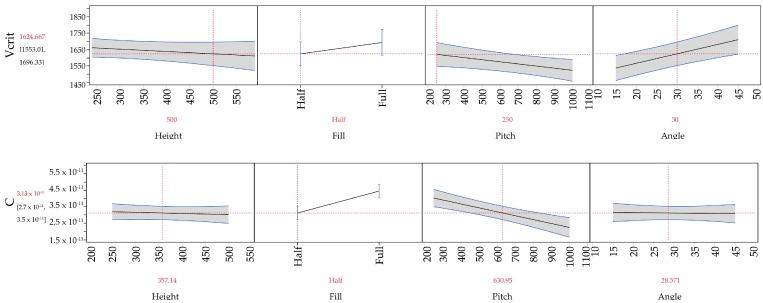
Response prediction profiles for the four factors varied in this experiment based on regression analysis for corona inception voltage (**top**) and for *C* (**bottom**). These plots in conjunction with the significance profiles from Figure 5 can be used to select an optimized geometry.

Angle, fill (half or full rank of spines), and separation were all found to be statistically significant (*p* < 0.05 ) factors affecting inception voltage, with tip angle being the dominant factor. Spine height was found to be statistically insignificant. For *C*, emitter fill and separation were found to be the only two significant factors. These models allow us to generate prediction plots for the quantitative effect of the various factors on the response; these are shown in Figure 6.

**Figure 7:**
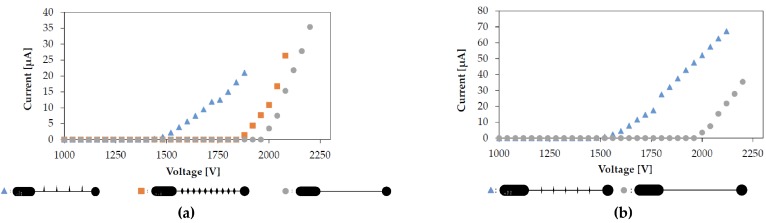
All tests performed with a gap distance of 500 μm and a collector grid separation of 500 μm. (**a**) The current-voltage characteristics of the lowest inception voltage configuration, highest *C* configuration, and a bare wire; (**b**) The current-voltage characteristics of the “optimized” wire, with separation of 750 μm, tip angle of 15°, height of 250 μm, and full fill. This electrode had an inception voltage of 1542 V and a *C* of 5.63 × 10^−11^ A/V^2^.

By following these trends we can identify the two geometries that should independently have the lowest inception voltage and the highest *C*. This is confirmed experimentally as shown in Figure 7. The lowest measured inception voltage is 1453 V and the highest measured *C* is 5.65 × 10^−11^ A/V^2^, as compared to the bare wire’s 1981 V and 7.30 × 10^−11^ A/V^2^. Noting that tip angle is the dominant driver of inception voltage and does not significantly affect *C* we can select an “optimized” design. At this point no weighting has been attached to the responses for a true optimization problem to be set up. The electrical characteristics of this geometry compared to the bare wire are also shown in Figure 7.

### 3.2. Loss Factor

Loss factor can be determined by plotting the theoretical electrostatic force (Equation (1)) versus the force measured using the grid outlet air flow. The slope of this line represents the fraction of theoretical force being produced as measured output force in the preferred direction. An ion mobility of 2 × 10^−4^ m^2^·V^−1^·s^−1^ and the nominal electrode gap distance were used in the theoretical force equation in all cases. Although that means loss factor in this case will also include deviation in ion mobility from this value, that error will presumably be a static offset and it is still a useful tool to examine trends. This method was previously explored in [12], where a loss factor term *β* was introduced to Equation (1).

**Figure 8:**
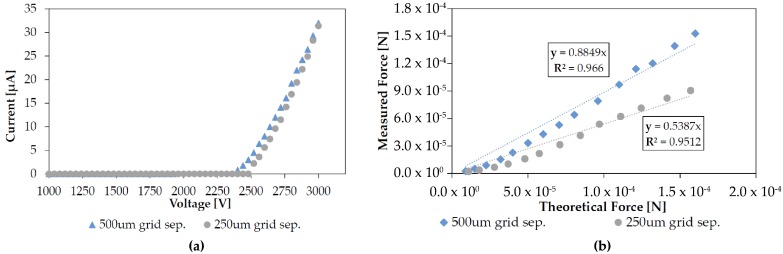
(**a**) Changing the grid wire separation from 500 μm to 250 μm had an insignificant effect on discharge characteristics with a bare wire at a 1 mm gap distance; (**b**) Decreasing the wire separation changed the loss factor by over 30%. Because there is no change in IV characteristics the increased losses can presumably be attributed to increased drag.

Grid wire separation is directly related to open area fraction. As shown in Figure 8, loss factor was found to be affected by this value without a change in discharge electrical characteristics for some values of separation. For a 1 mm electrode gap, discharge with a 750 μm wire separation grid was inconsistent and sparkover occurred before discharge for all larger separations. For a 500 mm gap, however, discharge was consistently measured using 500 μm wire separations. At this time it is unclear what causes this discrepancy in stable gap-separation ratio.

**Figure 9:**
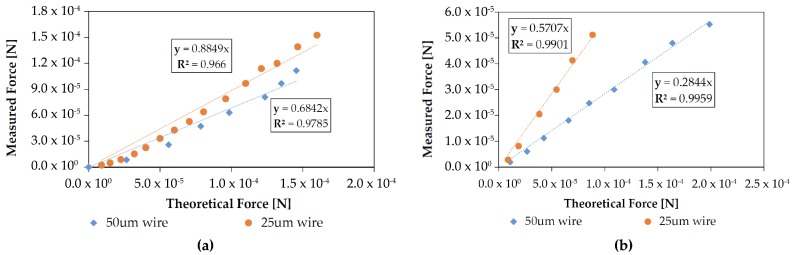
Both of these figures are for 500 μm grid wire separations with a bare wire as the emitter electrode. (**a**) Changing the grid wire width from 25 μm to 50 μm at a 1 mm electrode gap increased loss factor by about 20% without a significant effect on discharge current; (**b**) Changing the grid wire width from 25 μm to 50 μm at a 0.5 mm electrode gap increased loss factor by about 19% without a significant effect on discharge current.

Grid wire width was found to have a significant effect on loss factor. For a 500 μm wire separation grid, the open area fraction is about 91% for a 25 μm wire width and about 83% for a 50 μm width. This 8% change in open area fraction resulted in a 20% increase in measured loss factor, as shown in Figure 9.

**Figure 10:**
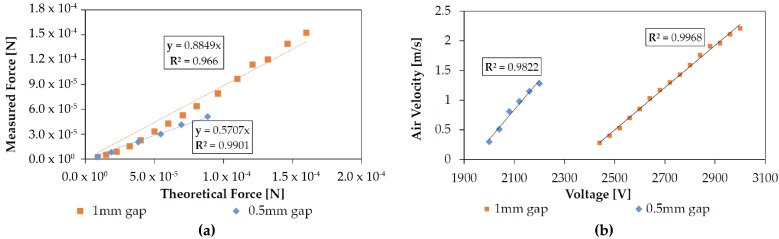
(**a**) Changing the electrode gap distance from 1 mm to 500 μm with all else held constant increased loss factor by over 30%. Test performed with a bare wire, a 500 μm grid wire separation, and a 25 μm wire width; (**b**) Measured outlet air velocity versus applied voltage for the two electrode gap distances. As expected by combining Equations (1) and (2) with the mass flow equation, the voltage-velocity relationship is linear above the corona onset voltage.

Loss factor was found to be a strong function of electrode gap, as shown in Figure 10. With all other factors constant a 50% decrease in electrode gap increased loss factor by over 30%.

### 3.3. Failure Modes

**Figure 11:**
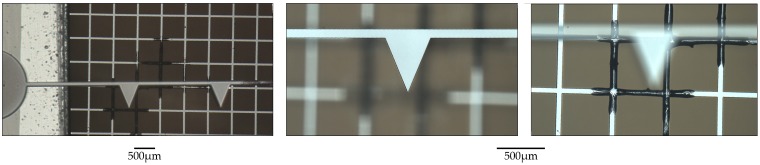
A typical failure mode of an electrode set. Permanent damage is sometimes observed following sparkover from a wire tip to the collector grid. This may be due to the relatively high current density following dielectric breakdown causing rapid Joule heating in the grid wires, or due to physical ablation during the high current breakdown. After a destructive event such as this, both electrodes were replaced despite there being no visible damage to the emitter wire.

Once the electric field reaches a critical magnitude dielectric breakdown occurs. The high voltage supply used during characterization has a current limit of 500 μA, which is typically reached during these sparkover events. The two most common catastrophic failure modes are for (1) the emitter wire to “pop” and break somewhere in the vicinity of the center or (2) for the collector grid to be physically damaged in the vicinity of the sparkover point. The latter case is shown in Figure 11.

## 4. Discussion and Conclusions

**Figure 12:**
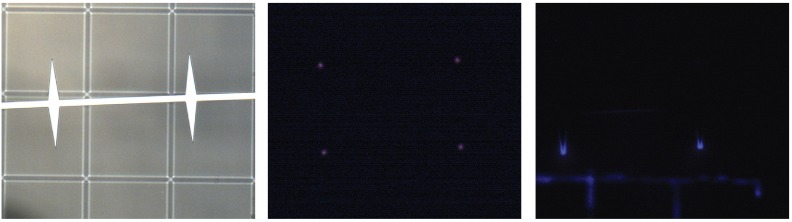
The corona discharge is characterized by a purple/violet glow visible in low light conditions. With the probe station door shut during electrical testing it was possible to take still images capturing the corona plasma. The first pane is the device with the light turned on, before testing. In the middle pane the device is at about 2500 V applied potential, where the plasma is clearly localized to the spine tips. The third pane is a still frame taken during a sustained breakdown event after the device had failed around 3000 V.

The fact that tip angle does not have a statistically significant effect on current generation seems to indicate that the corona plasma is highly localized; this is supported visually by the visible corona plasma being confined to the tips, as shown in Figure 12. This contrasts with [25], which noted an effect of the tip angle used for emission on corona discharge current. Further analysis of the dataset shows that there is a significant correlation (*p* = 0.01) between the number of tips and *C*. Because the height of the spines did not have a clear effect on either the inception voltage or the discharge current, it may be possible to design a new geometry that increases *C* without affecting onset potential; for example, a move from triangular spines to a triangle-on-a-post.

Increased corona inception voltage for small tip separation may be explained by a shielding effect similar to that noted in both field emission array literature and in multiple-needle corona discharge studies. Although we expect to see some coupling between minimum separation to avoid shielding and height of the tips, there is no evidence of that with the currently fabricated device geometries.

Although positive corona discharge is primarily driven by geometry and electrode material choice is unlikely to strongly affect operating voltage, the catastrophic failure mode sometimes exhibited may be related to material properties (e.g., resistivity). Metal emitter electrodes in sub-millimeter corona devices have their own unique failure modes, including mechanical yield [20]. Therefore instead of switching materials entirely a first step will be to investigate the effects of silicon doping concentration on device performance. Regardless, in an autonomous microrobot it is unlikely that the power circuitry would be able to supply current far in excess of the maximum operating point, perhaps negating this issue.

Holding all other factors constant and decreasing the electrode gap distance increased the loss factor by around 30%. We attribute this increase to two related primary reasons: increase in the ionization region to drift region ratio, and increase in the percentage of collisions imparting horizontal momentum components. The former will influence the effective *d* of Equation (1) and the latter will break the assumption that all collision energy generates force in the preferred direction.

As electrode gap is decreased the grid wire separation must also decrease to prevent them from influencing the emitter field. Decreasing the grid wire separation from 500 μm to 250 μm was found to increase loss factor by about 30%. Interestingly, this is roughly the same decrease in open area fraction (about 10%) that caused a 20% loss factor change when grid wire width was varied. This would imply that edge effects at the grid wires and not just obstructed area plays a role in drag through the grid; this theory is supported by literature, where drag through a micromesh decreased when edges were beveled [23].

Although the hybrid wire-needle design presented herein was successful in decreasing corona onset voltage, the current setup is not optimized for flow through the grid.

In conclusion, we have explored a method to lithographically define features that can be used to decrease operating voltage without unduly influencing discharge current. Further, we have attempted to test the limits of collector grid geometry scaling, demonstrating changes in loss factor of around 30% with no change in discharge current. Future work will investigate the limits of the scaling trends for lithographically defined emitter features in an effort to decrease operating voltage below 1000 V at an acceptable force loss factor; these electrodes will then be incorporated into future designs of flying ionocraft.

## Figures and Tables

**Table 1:** The electrode geometric factors and their values that were investigated in this work.

Emitter Wires				Collector Grids	
**Separation**	**Height**	**Angle**	**Fill**	**Separation**	**Width**
250	250	15	Half	250	10
500	500	30	Full	500	25
750	-	45	-	750	50
1000	-	-	-	1000	-
-	-	-	-	1250	-

## Abbreviations

The following abbreviations are used in this manuscript:

EHD	Electrohydrodynamic
MEMS	Micro electromechnical systems
DRIE	Deep reactive ion etch
IC	Integrated circuit
